# Supplementary material for: Methods used to assess outcome consistency in clinical studies: A literature-based evaluation
Source: PLoS One. 2020 Jul 8;15(7):e0235485. doi: 10.1371/journal.pone.0235485 (PMC7343158; doi:10.1371/journal.pone.0235485)
Supplement: S1 Fig — (DOCX) [file pone.0235485.s001.docx]

**S1 Fig** Study selection flow diagram

**Duffy 2017 [10], CoRe Outcomes in Women and Newborn health initiative website, Google search and the reference search*

**Included records**

**(N = 93)**

**Summary of the evaluation in a final Core Outcome Set report (N = 3)**

**Full reports of evaluation studies of outcome consistency
(N = 90)**

**Assessed records**

**(N = 237)**

**Excluded (N = 144) due to:**

- No publication available or no access to full text (N = 21)
- Core Outcome Set with no relevant details (N = 5)
- Focus only on a specific group of outcomes
  (N = 22)
- Focus on a single treatment (N = 2)
- Not relevant study objective (N = 61)
- Assessment of measurements (N = 20)
- Not relevant study design (N = 13)

**COMET database search**

**Jun 2018 (N = 189)**

**May 2019 (N = 41)**

**Other sources***

**(N = 53)**

**All records**

**(N = 283)**

**Duplicates**

**(N = 46)**
