# Supplementary material for: Methods used to assess outcome consistency in clinical studies: A literature-based evaluation
Source: PLoS One. 2020 Jul 8;15(7):e0235485. doi: 10.1371/journal.pone.0235485 (PMC7343158; doi:10.1371/journal.pone.0235485)
Supplement: S1 Text — (DOCX) [file pone.0235485.s002.docx]

**S1 Text** Data Collection Form – evaluation of outcome consistency in clinical studies (version 5.0)

| **PART I - General** | | | | | | | | | | | |
| --- | --- | --- | --- | --- | --- | --- | --- | --- | --- | --- | --- |
| **Date** (dd/mm/yy) |  | | | | **Reviewer ID** | |  | | | | |
| **Source of publication  Journal yy;vol(iss):pp** |  | | | | **Study ID  (Author + Year)** | |  | | | | |
| **Title** |  | | | | | | | | | | |
| **Systematic review in a title?** | | | | | □ Yes □ No | | | | | | |
| **PART II – Study characteristics** | | | | | | | | | | | |
| **Medical speciality** |  | | | | **Disease area** | | | |  | | |
| **Review aim** |  | | | | | | | | | | |
| **Inclusion criteria** |  | | | | | | | | | | |
| **Exclusion criteria** |  | | | | | | | | | | |
| **PART III – Systematic review methods** | | | | | | | | | | | |
| **Protocol in a public domain?** | | □ Yes □ No | | |  | | | | | | |
|  |  | If yes, is it | | | □ PROSPERO  □ COMET database  □ Published protocol of the review  □ Published protocol of core outcome set | | | | | | |
| ***2.1 Details of the systematic review*** | | | | | | | | | | | |
| **Included study design(s)**  *(tick all relevant)* | □ Systematic reviews  □ Randomised Controlled Trials (RCTs)  □ Only full-scale RCTs  □ Full-scale and pilot / feasibility RCTs  □ Non-RCT designs – non-RCT design (cohorts, case-controls,  case-series, case-reports, registers, etc.) | | | | | | | | | | |
| *If systematic reviews were included, were they used as* | | | | | *□ source of primary studies*  *□ source of outcomes* | | | | | | |
| ***2.1 Details of the systematic review (cont.)*** | | | | | | | | | | | |
| **Bibliographic database search** | **The time frame of search** | | |  | | **Was time limit applied?** | | | | *□ Yes □ No* | |
|  | **Number of database** | | |  | | **Type** | | □ *General only*  □ *General & specialist* | | | |
| *Other sources of information such as clinical trial registers?* | | | | *□ Yes □ No* | | *If yes, how many?* | | | | |  |
| *Example of databases: General - Medline, Embase, Cochrane CENTRAL, Scopus; Specialist - CINAHL, PsycINFO* | | | | | | | | | | | |
| ***2.2 AMSTAR 2*** *(Shea et al. 2017* *21;358:j4008)* | | | | | | | | | | | |
| Q1. Did the research questions and inclusion criteria for the review include the components of PICO? | | | | | | *□ Yes □ No □ Can’t answer* | | | | | |
| Q2. Did the report of the review contain an explicit statement that the review methods were established prior to the conduct of the review and did the report justify any significant deviations from the protocol | | | | | | *□ Yes □ No □ Can’t answer* | | | | | |
| Q3. Did the review authors explain their selection of the study designs for inclusion in the review? | | | | | | *□ Yes □ No □ Can’t answer* | | | | | |
| Q4. Did the review authors use a comprehensive literature search strategy? | | | | | | *□ Yes □ No □ Can’t answer* | | | | | |
| Q5. Did the review authors perform study selection in duplicate? | | | | | | *□ Yes □ No □ Can’t answer* | | | | | |
| Q6. Did the review authors perform data extraction in duplicate? | | | | | | *□ Yes □ No □ Can’t answer* | | | | | |
| Q7. Did the review authors provide a list of excluded studies and justify the exclusions | | | | | | *□ Yes □ No □ Can’t answer* | | | | | |
| Q8. Did the review authors describe the included studies in adequate detail? | | | | | | *□ Yes □ No □ Can’t answer* | | | | | |
| Q9. Did the review authors assessed the quality of included studies? | | | | | | *□ Yes □ No □ Can’t answer* | | | | | |
| *Q.10 - 16 Not applicable* | | | | | | | | | | | |
| ***2.3 Studies in the review*** | | | | | | | | | | | |
| **Number of included studies** | | | Primary  (RCT, non RCT) |  | | Secondary  (literature reviews) | | | | |  |
| **For primary studies** | | | | | | | | | | | |
| *Was a distinction made between primary and subsequent publications from the same trial?* | | | | | | *□ Yes □ No □ Can’t answer* | | | | | |
| **Total number of participants included in primary studies** | | | | | |  | | | | | |
| **Number of intervention groups** | | | | | |  | | | | | |

| **PART IV – Outcomes** | | | | |
| --- | --- | --- | --- | --- |
| ***4.1 Methods*** | | | | |
| ***How the primary outcomes were identified?*** | | | | |
| *□* Had to be clearly defined as „primary” *□* Outcomes used in power calculation □ Can’t answer | | | | |
| **Did the reviewers included outcomes reported** | | | | |
| *□* Only in methods section *□* Only in the results section *□* Anywhere in the publication  □ Can’t answer | | | | |
| **Was the quality of outcome reporting assessed?** | | | *□ Yes □ No* | |
| *If yes, what tool was used* | *□* Questionnaire from Harman et al. 2013 (MOMENT study)  *□* ORBIT  *□* CONSORT checklist for outcome reporting  *□* Other*, if selected, pleas e specify* | | | |
| ***4.2 Results*** | | | | |
| **Number of identified outcomes** | |  | | |
| **Number of outcome groups/domains** | |  | | |
| **Presentation** | | *□* Number of studies reporting outcome to the number of all included studies (n/N, %)  *□* Matrix (study by outcome tabulation)  *□* Narrative summary  □ Other | | |
| ***4.3 Study conclusion*** | | | | |
| **In discussion the authors concluded that they identified variation in outcome reporting or that the core outcome set is needed** | | | | *□ Yes*  *□ No*  *□ Unclear* |
| *If yes, what was the justification supporting this claim?* | | | | |
|  | | | | |
