# Supplementary material for: Methods used to assess outcome consistency in clinical studies: A literature-based evaluation
Source: PLoS One. 2020 Jul 8;15(7):e0235485. doi: 10.1371/journal.pone.0235485 (PMC7343158; doi:10.1371/journal.pone.0235485)
Supplement: S1 Table — (DOCX) [file pone.0235485.s003.docx]

**S1 Table** Description of how A MeaSurement Tool to Assess systematic Reviews (AMSTAR 2) items were applied

| **No** | **Question** | **Yes** | **No** | **Unclear** | **Not Applicable** |
| --- | --- | --- | --- | --- | --- |
| **1** | Did the research questions and inclusion criteria for the review include the components of PICO? | Elements of PICO structure identifiable | PICO elements unclear | Insufficient detail | Domain always assessed |
| **2** | Did the report of the review **contain an explicit statement that the review methods were established prior** to conduct of the review and did the report justify any significant deviations from the protocol | Reference to a prospectively developed and registered protocol available in a public domain or available on request. | Protocol not mentioned. | A protocol is mentioned in the text but without any reference | Domain always assessed |
| **3** | Did the review authors explain their selection of the study designs for inclusion in the review? | A clear justification given / part of study objective (e.g. in RCTs) | Lack of any justification. | Domain always assessed Yes or No | Domain always assessed |
| **4** | Did the review authors use a comprehensive literature search strategy? | More than 3 databases comprehensive search terms, no language restrictions | One or two databases searched **OR** language restriction | Three databases searched **AND** time limit or basic search terms | The domain was not assessed, if literature search was not performed |
| **5** | Did the review authors perform study selection in duplicate? | Clear statement of study selection in duplicate present | Statement that only one reviewer assessed studies eligibility | No statement in the text | The domain was not assessed, if the study relied on available literature review(s). |
| **6** | Data extract in duplicate? | Clear statement of data extraction in duplicate present | Statement that only one reviewer extracted data | No statement in the text | Domain always assessed |
| **7** | Did the review authors provide a list of excluded studies and justify the exclusions? | A complete list of potentially relevant studies with justification for the exclusion of individual studies given. | Lack exclusions list or only a summary of exclusion reasons on study selection flow diagram | List of excluded studies given but without supporting justifications | The domain was not assessed, if literature search was not performed or study relied on already existing systematic review |
| **8** | Did the review authors describe the included studies in adequate detail? | A table of study characteristics for individual studies (main manuscript or appendix) | A short summary of studies in the text or a summary table | A table of study characteristics for individual studies (main text or appendix) lacking key details (PICO) | The domain was not assessed, if study relied on already existing systematic review or only a short summary of outcome assessment was available (COS reports) |
| **9** | Was study quality appraised? | Details of study quality appraisal given | No information on study quality appraisal | Domain always assessed Yes, No or Not applicable | The domain was not assessed, if only a short summary of outcome assessment was available (COS reports) |
