# Supplementary material for: Methods used to assess outcome consistency in clinical studies: A literature-based evaluation
Source: PLoS One. 2020 Jul 8;15(7):e0235485. doi: 10.1371/journal.pone.0235485 (PMC7343158; doi:10.1371/journal.pone.0235485)
Supplement: S3 Table — (DOCX) [file pone.0235485.s005.docx]

**S3 Table** Detailed characteristics of included evaluation studies of outcome consistency

| **Study ID** | **Medical speciality** | **Called a systematic review by the authors? (if not, term given)** | **Included designs** | **No. of included studies** | **Inclusion of main and secondary publications?** | **Protocol in the public domain?** | **Distinction between primary and secondary outcomes?** | **Identification of primary outcome** | **Outcome extraction (location)** | **No of identified outcomes** |
| --- | --- | --- | --- | --- | --- | --- | --- | --- | --- | --- |
| Agha  2016 [1] | Plastic, reconstructive and aesthetic surgery | Yes | Any design | 35 | Yes | Yes (PROSPERO, publication) | No | N/A | Entire publication | 35 |
| Al Jabri  2019 [2] | Otorhinolaryngology | No  (a review/ literature review) | Syst rev, Clinical Trials & Observational | 143 | Unclear | Yes (Institutional website) | Yes | Unclear | Unclear | A:42 OMD:68 S:33 |
| Al Wattar  2015 [3] | Women’s Health | Yes | Clinical Trials & Observational | 232 | No | COMET | No | N/A | Unclear | 232 |
| Alkhaffaf  2018 [4] | Oncology | Yes | Syst rev & RCTs | 32 | Yes | publication | Yes | Clearly defined OR power calculation | Unclear | 32 |
| Allin  2016 [5] | Pediatrics & Neonatology | Yes | Clinical Trials & Observational | 65 | No | PROSPERO | Yes | Unclear | Unclear | G: 30  HD: 35 |
| Andersen 2018 [6] | Neurology | Yes | Full-scale and pilot / feasibility RCTs | 129 | Unclear | COMET | Yes | Clearly defined | Unclear | 129 |
| Ardestani 2017 [7] | Endocrinology | Yes | RCTs | 231 | No | PROSPERO | Yes | Clearly defined | Unclear | 231 |
| Begley  2014 [8] | Women’s Health | Yes | Syst rev & RCTs | 33 | Yes | No | Yes | Clearly defined OR power calculation | Entire publication | 33 |
| Benstoem 2015 [9] | Heart, circulation, blood | Yes | Syst rev | N/A | N/A | COMET | No | N/A | Unclear | 15 |
| **Study ID** | **Medical speciality** | **Described as a systematic review?** | **Included designs** | **No. of included studies** | **Inclusion of main and secondary publications?** | **Protocol in the public domain?** | **Distinction between primary and secondary outcomes?** | **Identification of primary outcome** | **Outcome extraction (location)** | **No of identified outcomes** |
| Bhangu  2015 [10] | Gastroenterology | No  (an analysis of outcomes) | Meta-analysis & RCTs | 47 | Yes | COMET | Yes | Unclear | Unclear | 40 |
| Blackwood 2014 [11] | Anaesthesiology | No  (a review/ literature review) | RCTs | 66 | Yes | No | Yes | Unclear | Unclear | 66 |
| Blencowe 2012b [12] | Oncology | Yes | Clinical Trials & Observational | 122 | Unclear | No | No | N/A | Unclear | 122 |
| Bonnett  2015 [13] | Infectious disease | Yes | Full-scale RCTs | 55 | Unclear | No | Yes | Unclear | Unclear | 55 |
| Bonnett  2018 [14] | Infectious disease | Yes | Clinical Trials | 248 | Unclear | Available on request | Yes | Clearly defined | Unclear | 248 |
| Chari  2015 [15] | Trauma | Yes | Clinical Trials & Observational | 102 | Unclear | PROSPERO | Yes | Unclear | Methods only | 102 |
| Chiu  2014 [16] | Ophthalmology | Yes | Unclear | 56 | Unclear | No | No | N/A | Unclear | 56 |
| Dapuzzo  2011 [17] | Women’s Health | No  (a review/ literature review) | Clinical Trials | 294 | Unclear | No | No | N/A | Unclear | 294 |
| Davies  2016 [18] | Neurology | Yes | Any design | 108 | Unclear | PROSPERO | Yes | Unclear | Results only | 108 |
| de Mattos Lourenco 2019a [19] | Women’s Health | Yes | RCTs | 51 | Yes | PROSPERO | Yes | Clearly defined | Unclear | 51 |
| **Study ID** | **Medical speciality** | **Described as a systematic review?** | **Included designs** | **No. of included studies** | **Inclusion of main and secondary publications?** | **Protocol in the public domain?** | **Distinction between primary and secondary outcomes?** | **Identification of primary outcome** | **Outcome extraction (location)** | **No of identified outcomes** |
| de Mattos Lourenco 2019b [20] | Women’s Health | Yes | RCTs | 32 | Yes | PROSPERO | Yes | Clearly defined | Unclear | 32 |
| Deckert  2016 [21] | Anaesthesiology | Yes | Syst rev, Clinical Trials & Observational | 70 | Yes | COMET | Yes | Unclear | Results only | 70 |
| Denniston 2015 [22] | Ophthalmology | Yes | Clinical Trials | 104 | N/A | No | Yes | Clearly defined | Methods only | 104 |
| Dos Santos 2018* [23] | Women’s Health | No  (a review/ literature review) | Syst rev | 32 | Unclear | COMET | Unclear | N/A | Unclear | 32 |
| Duffy  2017 [24] | Women’s Health | Yes | RCTs | 79 | Unclear | PROSPERO, publication | Yes | Unclear | Unclear | 79 |
| Duncan Millar 2019 [25] | Neurology | No  (a systematic exploration) | Syst rev & Clinical Trials | 243 | Unclear | No | No | N/A | Unclear | 243 |
| Durnea  2018 [26] | Women’s Health | Yes | RCTs | 68 | Yes | PROSPERO | Yes | Clearly defined | Unclear | 68 |
| Egan  2017* [27] | Women’s Health | Yes | Syst rev, Clinical Trials & Observational | 33 | Unclear | COMET, publication | Unclear | N/A | Entire publication | 33 |
| Eleftheriadou 2012 [28] | Dermatology | Yes | Full-scale RCTs | 54 | Unclear | No | Yes | Clearly defined | Entire publication | 54 |
| Faggion  2010 [29] | Oncology | Yes | Clinical Trials | 14 | Yes | No | Yes | Clearly defined | Entire publication | 14 |
| **Study ID** | **Medical speciality** | **Described as a systematic review?** | **Included designs** | **No. of included studies** | **Inclusion of main and secondary publications?** | **Protocol in the public domain?** | **Distinction between primary and secondary outcomes?** | **Identification of primary outcome** | **Outcome extraction (location)** | **No of identified outcomes** |
| Fish  2018 [30] | Oncology | Yes | Syst rev, Clinical Trials & Observational | 101 | Yes | PROSPERO | No | N/A | Entire publication | 101 |
| Fitzpatrick 2010 [31] | Mental health | Yes | Clinical Trials & Observational | 308 | Yes | No | No | N/A | Unclear | 308 |
| Gethin  2015 [32] | Dermatology | Yes | RCTs | 102 | Unclear | No | Yes | Unclear | Unclear | 102 |
| Glynne-Jones 2017 [33] | Oncology | No  (a review/ literature review) | Full-scale RCTs | 6 | Yes | No | Yes | Unclear | Unclear | 6 |
| Goncalves 2018 [34] | Oncology | Yes | Clinical Trials & Observational | 130 | Yes | Available on request | No | N/A | Entire publication | 130 |
| Hall  2016 [35] | Otorhinolaryngology | Yes | Syst rev, Clinical Trials & Observational | 228 | Yes | PROSPERO, publication | Yes | Clearly defined | Entire publication | 228 |
| Hancox  2019 [36] | Trauma | Yes | RCTs | 27 | Unclear | No | Yes | Unclear | Unclear | 27 |
| Hanson  2019 [37] | Nephrology | No  (an electronic database search) | RCTs & Observational | 268 | Unclear | No | No | N/A | Unclear | 268 |
| Harman  2017 [38] | Endocrinology | Yes | Full-scale RCTs | 138 | N/A | No | Yes | Clearly defined | Methods only | 138 |
| **Study ID** | **Medical speciality** | **Described as a systematic review?** | **Included designs** | **No. of included studies** | **Inclusion of main and secondary publications?** | **Protocol in the public domain?** | **Distinction between primary and secondary outcomes?** | **Identification of primary outcome** | **Outcome extraction (location)** | **No of identified outcomes** |
| Hatemi  2014 [39] | Rheumatology | Yes | Clinical Trials & Observational | 249 | Unclear | No | No | N/A | Unclear | 249 |
| Herman  2016 [40] | Women’s Health | No  (a review/ literature review) | Syst rev & RCTs | 92 | Unclear | No | Yes | Clearly defined OR power calculation | Methods only | 92 |
| Hirsch  2016 [41] | Women’s Health | Yes | RCTs | 54 | Unclear | COMET | Yes | Unclear | Unclear | 54 |
| Hopkins  2015 [42] | Endocrinology | Yes | Clinical Trials & Observational | 90 | Yes | Available on request | Yes | Clearly defined OR power calculation | Methods only | 90 |
| Horton  2015 [43] | General medicine | No  (a review/ literature review) | Observational | 7 | Unclear | No | No | N/A | Unclear | 7 |
| Hujoel  1995 [44] | Dentistry & oral health | No  (a survey) | RCTs | 82 | Yes | No | No | N/A | Unclear | 82 |
| Hussain  2016 [45] | Surgery | No  (not clearly specified probably a review) | RCTs | 182 | Yes | No | Yes | Clearly defined OR power calculation | Methods only | 182 |
| Ismail  2015 [46] | Otorhinolaryngology | Yes | Syst rev | 21 | N/A | No | Yes | Clearly defined | Methods only | 21 |
| Jerosch-Herold  2006 [47] | Neurology | Yes | RCTs & quasi-RCTs | 28 | Unclear | No | Yes | Unclear | Unclear | 28 |
| **Study ID** | **Medical speciality** | **Described as a systematic review?** | **Included designs** | **No. of included studies** | **Inclusion of main and secondary publications?** | **Protocol in the public domain?** | **Distinction between primary and secondary outcomes?** | **Identification of primary outcome** | **Outcome extraction (location)** | **No of identified outcomes** |
| Kapadia  2016 [48] | Neurology | Yes | Clinical Trials & Observational | 104 | Unclear | COMET | Yes | Clearly defined | Entire publication | 104 |
| Kaufman  2016 [49] | Public health | No  (outcome mapping) | Clinical Trials | 112 | Unclear | No | No | N/A | Unclear | 112 |
| Koot  2018 [50] | Women’s Health | Yes | RCTs | 34 | Unclear | COMET | Yes | Unclear | Unclear | 34 |
| Kuizenga-Wessel  2016 [51] | Gastroenterology | Yes | Syst Rev & RCTs | 45 | Unclear | No | Yes | Unclear | Unclear | 45 |
| Malinowski 2019 [52] | Heart, circulation, blood | Yes | RCTs | 33 | Unclear | PROSPERO | Yes | Unclear | Entire publication | 33 |
| Marks  2013 [53] | Orthopaedics | Yes | Clinical Trials & Observational | 316 | Unclear | Other register | No | N/A | Entire publication | 316 |
| Matvienko Sikar 2019 [54] | Pediatrics & Neonatology | Yes | Any design | 126 | Unclear | PROSPERO, publication | Yes | Clearly defined | Entire publication | 126 |
| Meher  2014 [55] | Women’s Health | Yes | RCTs | 103 | Yes | No | Yes | Clearly defined | Methods only | 103 |
| Meher  2019* [56] | Women’s Health | Yes | RCTs | 121 | Unclear | No | Unclear | N/A | Entire publication | 121 |
| Mellor  2018 [57] | Gastroenterology | Yes | RCTs | 51 | Unclear | PROSPERO, publication | No | N/A | Unclear | 51 |
| Metryka  2019 [58] | Otorhinolaryngology | Yes | Lit rev, Clinical Trials & Observational | 18 | Unclear | No | No | N/A | Unclear | 18 |
| **Study ID** | **Medical speciality** | **Described as a systematic review?** | **Included designs** | **No. of included studies** | **Inclusion of main and secondary publications?** | **Protocol in the public domain?** | **Distinction between primary and secondary outcomes?** | **Identification of primary outcome** | **Outcome extraction (location)** | **No of identified outcomes** |
| Opondo  2014 [59] | Urology | No  (a review/ literature review) | RCTs | 83 | Unclear | No | No | N/A | Unclear | 83 |
| Page  2015 [60] | Orthopaedics | Yes | Clinical Trials | 171 | Yes | COMET | Yes | Clearly defined | Unclear | 171 |
| Page  2018 [61] | Orthopaedics | No  (a review/ literature review) | RCTs & quasi-RCTs | 409 | Unclear | No | No | N/A | Unclear | 409 |
| Pergialiotis 2018 [62] | Women’s Health | Yes | RCTs | 48 | Unclear | PROSPERO | Yes | Clearly defined | Unclear | 48 |
| Perry  2018 [63] | Women’s Health | Yes | Clinical Trials & Observational | 100 | Unclear | PROSPERO | Yes | Unclear | Entire publication | 100 |
| Potter  2011 [64] | Plastic, reconstructive and aesthetic surgery | Yes | Clinical Trials & Observational | 134 | Unclear | No | No | N/A | Unclear | 134 |
| Rahn  2011 [65] | Women’s Health | Yes | Full-scale and pilot / feasibility RCTs | 79 | Yes | No | Yes | Clearly defined OR power calculation | Unclear | 79 |
| Rodgers  2014 [66] | Orthopaedics | Yes | RCTs | 33 | Unclear | No | No | N/A | Unclear | 33 |
| Rogozinska 2017 [67] | Women’s Health | Yes | Full-scale and pilot / feasibility RCTs | 66 | Yes | No | Yes | Clearly defined | Results only | 66 |
| Ronsch  2019 [68] | Dermatology | Yes | Clinical Trials | 61 | Yes | No | Yes | Unclear | Entire publication | 61 |
| **Study ID** | **Medical speciality** | **Described as a systematic review?** | **Included designs** | **No. of included studies** | **Inclusion of main and secondary publications?** | **Protocol in the public domain?** | **Distinction between primary and secondary outcomes?** | **Identification of primary outcome** | **Outcome extraction (location)** | **No of identified outcomes** |
| Ross  2016 [69] | Pediatrics & Neonatology | Yes | Syst rev & RCTs | 8 | Unclear | COMET | Yes | Clearly defined OR power calculation OR named in the trial aim | Entire publication | 8 |
| Ross  2018 [70] | Anaesthesiology | Yes | Clinical Trials & Observational | 152 | N/A | COMET | Yes | Clearly defined | Methods only | 152 |
| Rubin  2016 [71] | Gastroenterology | Yes | Clinical Trials | 11 | Unclear | PROSPERO | Yes | Clearly defined | Unclear | 11 |
| Sadownik 2018 [72] | Women’s Health | Yes | RCTs & Observational | 33 | Unclear | PROSPERO | Yes | Unclear | Methods only | 33 |
| Sautenet  2016 [73] | Nephrology | Yes | Syst rev | 66 | N/A | No | Yes | Clearly defined | Entire publication | 66 |
| Sautenet  2018a [74] | Nephrology | Yes | Syst rev & RCTs | 397 | Yes | No | Yes | Clearly defined | Unclear | 397 |
| Sautenet  2018b [75] | Nephrology | Yes | Syst rev & RCTs | 362 | Yes | No | Yes | Clearly defined | Unclear | 362 |
| Sharif  2015 [76] | Dentistry & oral health | Yes | Syst rev & Clinical Trials | 10 | Unclear | COMET | No | N/A | Unclear | 10 |
| Simpson  2013 [77] | Dermatology | Yes | Full-scale and pilot / feasibility RCTs | 28 | Unclear | Other register | Yes | Clearly defined OR first reported | Entire publication | 28 |
| **Study ID** | **Medical speciality** | **Described as a systematic review?** | **Included designs** | **No. of included studies** | **Inclusion of main and secondary publications?** | **Protocol in the public domain?** | **Distinction between primary and secondary outcomes?** | **Identification of primary outcome** | **Outcome extraction (location)** | **No of identified outcomes** |
| Sims  2017 [78] | Orthopaedics | No  (an analysis of studies) | Clinical Trials & Observational | 66 | N/A | COMET | Yes | Clearly defined | Methods only | 66 |
| Singendonk 2017 [79] | Gastroenterology | Yes | RCTs | 46 | Unclear | No | Yes | Clearly defined | Unclear | GER: 26  GERD: 20 |
| Sinha  2009 [80] | Pulmonology | No  (a review/ literature review) | RCTs | 159 | Yes | No | Yes | Clearly defined OR power calculation | Unclear | 159 |
| Smaıl-Faugeron 2013 [81] | Dentistry & oral health | Yes | RCTs | 47 | Yes | No | Yes | Clearly defined OR power calculation | Entire publication | 47 |
| Smith  2019 [82] | Women’s Health | Yes | RCTs | 208 | Unclear | PROSPERO | Yes | Clearly defined OR power calculation | Unclear | 208 |
| Taverny  2019 [83] | Trauma | Yes | RCTs | 170 | Yes | PROSPERO | Yes | Clearly defined | Unclear | 170 |
| Tirlapur  2014 [84] | Women’s Health | Yes | Syst rev & RCTs | 36 | Unclear | No | No | N/A | Unclear | 36 |
| Townsend 2019 [85] | Women’s Health | Yes | RCTs | 68 | Yes | PROSPERO | Yes | Unclear | Unclear | 68 |
| Tsichlakia 2014 [86] | Dentistry & oral health | Yes | RCTs | 133 | Unclear | No | Yes | Clearly defined OR power calculation | Entire publication | 133 |
| **Study ID** | **Medical speciality** | **Described as a systematic review?** | **Included designs** | **No. of included studies** | **Inclusion of main and secondary publications?** | **Protocol in the public domain?** | **Distinction between primary and secondary outcomes?** | **Identification of primary outcome** | **Outcome extraction (location)** | **No of identified outcomes** |
| Tsichlakia 2017 [87] | Dentistry & oral health | No  (a scoping review) | Any design | 40 | Yes | No | Yes | Clearly defined | Entire publication | 40 |
| van Tol  2018 [88] | Gastroenterology | Yes | Clinical Trials & Observational | 34 | Unclear | No | No | N/A | Unclear | 34 |
| Whistance 2013 [89] | Oncology | Yes | All prospective designs (incl. registers) | 194 | Unclear | No | Yes | Clearly defined OR power calculation | Entire publication | 194 |
| Whitehead 2015 [90] | Heart, circulation, blood | Yes | Full-scale and pilot / feasibility RCTs | 61 | Unclear | No | Yes | Power calculation | Unclear | 61 |
| Wilkinson 2016 [91] | Women’s Health | No  (a review of outcomes) | RCTs | 142 | Unclear | No | No | N/A | Results only | 142 |
| Wittkopa  2010 [92] | Infectious disease | No  (a review of outcomes) | RCTs | 51 | Unclear | No | Yes | Unclear | Unclear | 51 |
| Young  2019 [93] | Trauma | Yes | Full-scale and pilot / feasibility RCTs | 147 | Unclear | No | No | N/A | Unclear | 147 |

*Syst rev, Systematic reviews*

**summary on outcomes reported in the literature in a final core outcome set report*

1. Agha, R.A., et al., *The Need for Core Outcome Reporting in Autologous Fat Grafting for Breast Reconstruction.* Ann Plast Surg, 2016. **77**(5): p. 506-512.

2. Al Jabri, S., J. Kirkham, and F.J. Rowe, *Development of a core outcome set for amblyopia, strabismus and ocular motility disorders: a review to identify outcome measures.* BMC Ophthalmol, 2019. **19**(1): p. 47.

3. Al Wattar, B.H., et al., *Variation in the reporting of outcomes among pregnant women with epilepsy: a systematic review.* Eur J Obstet Gynecol Reprod Biol, 2015. **195**: p. 193-9.

4. Alkhaffaf, B., et al., *Reporting of outcomes in gastric cancer surgery trials: a systematic review.* BMJ Open, 2018. **8**(10): p. e021796.

5. Allin, B., et al., *Variability of outcome reporting in Hirschsprung's Disease and gastroschisis: a systematic review.* Scientific reports, 2016. **6**: p. 38969.

6. Andersen, C.R., et al., *A Systematic Review of Outcome Measures Employed in Aneurysmal Subarachnoid Hemorrhage (aSAH) Clinical Research.* Neurocrit Care, 2019. **30**(3): p. 534-541.

7. Khanpour Ardestani, S., et al., *Primary outcomes reporting in trials of paediatric type 1 diabetes mellitus: a systematic review.* BMJ Open, 2017. **7**(12): p. e014610.

8. Begley, C., et al., *Outcome measures in studies on the use of oxytocin for the treatment of delay in labour: a systematic review.* Midwifery, 2014. **30**(9): p. 975-982.

9. Benstoem, C., et al., *Evaluating outcomes used in cardiothoracic surgery interventional research: a systematic review of reviews to develop a core outcome set.* PLoS One, 2015. **10**(4): p. e0122204.

10. Bhangu, A., et al., *A detailed analysis of outcome reporting from randomised controlled trials and meta-analyses of inguinal hernia repair.* Hernia, 2015. **19**(1): p. 65-75.

11. Blackwood, B., et al., *How outcomes are defined in clinical trials of mechanically ventilated adults and children.* American Journal of Respiratory and Critical Care Medicine, 2014. **189**(8): p. 886-893.

12. Blencowe, N., et al., *Reporting of short-term clinical outcomes after esophagectomy: A systematic review.* Annals of Surgery, 2012: p. no pagination.

13. Bonnett, L.J. and G.R. Davies, *Quality of outcome reporting in phase II studies in pulmonary tuberculosis.* Trials, 2015. **16**: p. 518.

14. Bonnett, L.J., G. Ken-Dror, and G.R. Davies, *Quality of reporting of outcomes in phase III studies of pulmonary tuberculosis: a systematic review.* Trials, 2018. **19**(1): p. 134.

15. Chari, A., et al., *Core Outcomes and Common Data Elements in Chronic Subdural Hematoma: A Systematic Review of the Literature Focusing on Reported Outcomes.* J Neurotrauma, 2016. **33**(13): p. 1212-9.

16. Chiu, A., et al., *Standardising reported outcomes of surgery for intermittent exotropia-a systematic literature review.* Strabismus, 2014. **22**(1): p. 32-36.

17. Dapuzzo, L., et al., *Incomplete and inconsistent reporting of maternal and fetal outcomes in infertility treatment trials.* Fertil Steril, 2011. **95**(8): p. 2527-30.

18. Davies, B.M., et al., *Reported Outcome Measures in Degenerative Cervical Myelopathy: A Systematic Review.* PLoS One, 2016. **11**(8): p. e0157263.

19. de Mattos Lourenco, T., et al., *A systematic review on reporting outcomes and outcome measures in trials on synthetic mesh procedures for pelvic organ prolapse: Urgent action is needed to improve quality of research.* Neurourology and Urodynamics, 2019. **38**(2): p. 509-524.

20. de Mattos Lourenco, T., et al., *A systematic review of reported outcomes and outcome measures in randomized controlled trials on apical prolapse surgery.* International Journal of Gynecology and Obstetrics, 2019. **145**(1): p. 4-11.

21. Deckert, S., et al., *A systematic review of the outcomes reported in multimodal pain therapy for chronic pain.* Eur J Pain, 2016. **20**(1): p. 51-63.

22. Denniston, A., et al., *Heterogeneity of primary outcome measures used in clinical trials of treatments for intermediate, posterior, and panuveitis.* Orphanet Journal of Rare Diseases, 2015. **10**(1): p. no pagination.

23. Dos Santos, F., et al., *Development of a core outcome set for trials on induction of labour: an international multistakeholder Delphi study.* BJOG, 2018. **125**(13): p. 1673-1680.

24. Duffy, J., et al., *Outcome reporting across randomised controlled trials evaluating therapeutic interventions for pre-eclampsia.* BJOG, 2017. **124**(12): p. 1829-1839.

25. Duncan Millar, J., et al., *Outcome measures in post-stroke arm rehabilitation trials: do existing measures capture outcomes that are important to stroke survivors, carers, and clinicians?* Clin Rehabil, 2019. **33**(4): p. 737-749.

26. Durnea, C.M., et al., *A systematic review of outcome and outcome-measure reporting in randomised trials evaluating surgical interventions for anterior-compartment vaginal prolapse: a call to action to develop a core outcome set.* Int Urogynecol J, 2018. **29**(12): p. 1727-1745.

27. Egan, A.M., et al., *A core outcome set for studies evaluating the effectiveness of prepregnancy care for women with pregestational diabetes.* Diabetologia, 2017. **60**(7): p. 1190-1196.

28. Eleftheriadou, V., et al., *Which outcomes should we measure in vitiligo? Results of a systematic review and a survey among patients and clinicians on outcomes in vitiligo trials.* British Journal of Dermatology, 2012. **167**(4): p. 804-814.

29. Faggion, C.M., Jr., S. Listl, and Y.K. Tu, *Assessment of endpoints in studies on peri-implantitis treatment--a systematic review.* J Dent, 2010. **38**(6): p. 443-50.

30. Fish, R., et al., *Systematic review of outcome measures following chemoradiotherapy for the treatment of anal cancer (CORMAC).* Colorectal Dis, 2018. **20**(5): p. 371-382.

31. Fitzpatrick, R., et al., *A systematic review of outcome measures used in forensic mental health research with consensus panel opinion.* Health Technology Assessment, 2010. **14**(18): p. 1-108.

32. Gethin, G., F. Killeen, and D. Devane, *Heterogeneity of wound outcome measures in RCTs of treatments for VLUs: a systematic review.* J Wound Care, 2015. **24**(5): p. 211-2, 214, 216 passim.

33. Glynne-Jones, R., et al., *Clinical endpoints in trials of chemoradiation for patients with anal cancer.* Lancet Oncol, 2017. **18**(4): p. e218-e227.

34. Goncalves, A.C., et al., *Evaluating physical activity in dementia: a systematic review of outcomes to inform the development of a core outcome set.* Age Ageing, 2018. **47**(1): p. 34-41.

35. Hall, D., et al., *Systematic review of outcome domains and instruments used in clinical trials of tinnitus treatments in adults.* Trials, 2016. **17**(1): p. no pagination.

36. Hancox, J., et al., *Patient-centred outcomes for prehospital trauma trials: A systematic review and patient involvement exercise.* Trauma (United Kingdom), 2019. **21**(4): p. 259-271.

37. Hanson, C.S., et al., *Informative for Decision Making? The Spectrum and Consistency of Outcomes After Living Kidney Donation Reported in Trials and Observational Studies.* Transplantation, 2019. **103**(2): p. 284-290.

38. Harman, N.L., et al., *SCORE-IT (Selecting Core Outcomes for Randomised Effectiveness trials In Type 2 diabetes): a systematic review of registered trials.* Trials, 2017. **18**(1): p. 597.

39. Hatemi, G., et al., *Outcome Measures Used in Clinical Trials for Behcet Syndrome: A Systematic Review.* Journal of Rheumatology, 2014. **41**(3): p. 599-612.

40. Herman, M.C., et al., *Choice of primary outcomes evaluating treatment for heavy menstrual bleeding.* BJOG, 2016. **123**(10): p. 1593-8.

41. Hirsch, M., et al., *Variation in outcome reporting in endometriosis trials: a systematic review.* Am J Obstet Gynecol, 2016. **214**(4): p. 452-64.

42. Hopkins, J.C., et al., *Outcome reporting in bariatric surgery: an in-depth analysis to inform the development of a core outcome set, the BARIACT Study.* Obes Rev, 2015. **16**(1): p. 88-106.

43. Horton, L., T. Duffy, and C.R. Martin, *Assessing outcomes of alcohol-related brain damage (ARBD): What should we be measuring?* Drugs-Education Prevention and Policy, 2015. **22**(2): p. 151-159.

44. Hujoel, P.P. and T.A. DeRouen, *A survey of endpoint characteristics in periodontal clinical trials published 1988-1992, and implications for future studies.* J Clin Periodontol, 1995. **22**(5): p. 397-407.

45. Hussain, S. and S.R. Knight, *Quality and consistency of outcome reporting in clinical trials of immunosuppression in renal transplantation.* Clin Transplant, 2016. **30**(11): p. 1440-1448.

46. Ismail, R., A. Azuara-Blanco, and C.R. Ramsay, *Outcome Measures in Glaucoma: A Systematic Review of Cochrane Reviews and Protocols.* J Glaucoma, 2015. **24**(7): p. 533-8.

47. Jerosch-Herold, C., et al., *A systematic review of outcomes assessed in randomized controlled trials of surgical interventions for carpal tunnel syndrome using the International Classification of Functioning, Disability and Health (ICF) as a reference tool.* BMC Musculoskeletal Disorders, 2006. **7**: p. no pagination.

48. Kapadia, M.Z., et al., *A Core Outcome Set for Children With Feeding Tubes and Neurologic Impairment: A Systematic Review.* Pediatrics, 2016. **138**(1).

49. Kaufman, J., et al., *Outcomes mapping study for childhood vaccination communication: too few concepts were measured in too many ways.* J Clin Epidemiol, 2016. **72**: p. 33-44.

50. Koot, M.H., et al., *Variation in hyperemesis gravidarum definition and outcome reporting in randomised clinical trials: a systematic review.* BJOG, 2018. **125**(12): p. 1514-1521.

51. Kuizenga-Wessel, S., et al., *Reporting on Outcome Measures of Functional Constipation in Children-A Systematic Review.* J Pediatr Gastroenterol Nutr, 2016. **62**(6): p. 840-6.

52. Malinowski, A.K., et al., *Reported Outcomes in Perinatal Iron Deficiency Anemia Trials: A Systematic Review.* Gynecol Obstet Invest, 2019. **84**(5): p. 417-434.

53. Marks, M., et al., *Outcome measures and their measurement properties for trapeziometacarpal osteoarthritis: a systematic literature review.* J Hand Surg Eur Vol, 2013. **38**(8): p. 822-38.

54. Matvienko-Sikar, K., et al., *Developing a core outcome set for childhood obesity prevention: A systematic review.* Matern Child Nutr, 2018: p. e12680.

55. Meher, S. and Z. Alfirevic, *Choice of primary outcomes in randomised trials and systematic reviews evaluating interventions for preterm birth prevention: a systematic review.* BJOG, 2014. **121**(10): p. 1188-94; discussion 1195-6.

56. Meher, S., et al., *Core outcome sets for prevention and treatment of postpartum haemorrhage: an international Delphi consensus study.* BJOG, 2019. **126**(1): p. 83-93.

57. Mellor, K., D. Hind, and M.J. Lee, *A systematic review of outcomes reported in small bowel obstruction research.* J Surg Res, 2018. **229**: p. 41-50.

58. Metryka, A., et al., *Toward a Core Outcome Set for Head, Neck, and Respiratory Disease in Mucopolysaccharidosis Type II: Systematic Literature Review and Assessment of Heterogeneity in Outcome Reporting.* Journal of Inborn Errors of Metabolism and Screening, 2019. **7**: p. no pagination.

59. Opondo, D., et al., *Standardization of patient outcomes reporting in percutaneous nephrolithotomy.* J Endourol, 2014. **28**(7): p. 767-74.

60. Page, M.J., et al., *Core domain and outcome measurement sets for shoulder pain trials are needed: systematic review of physical therapy trials.* J Clin Epidemiol, 2015. **68**(11): p. 1270-81.

61. Page, M.J., et al., *Outcome Reporting in Randomized Trials for Shoulder Disorders: Literature Review to Inform the Development of a Core Outcome Set.* Arthritis Care Res (Hoboken), 2018. **70**(2): p. 252-259.

62. Pergialiotis, V., et al., *Do we need a core outcome set for childbirth perineal trauma research? A systematic review of outcome reporting in randomised trials evaluating the management of childbirth trauma.* BJOG, 2018. **125**(12): p. 1522-1531.

63. Perry, H., et al., *Outcome reporting across randomized trials and observational studies evaluating treatments for twin-twin transfusion syndrome: systematic review.* Ultrasound Obstet Gynecol, 2018. **52**(5): p. 577-585.

64. Potter, S., et al., *Reporting clinical outcomes of breast reconstruction: a systematic review.* J Natl Cancer Inst, 2011. **103**(1): p. 31-46.

65. Rahn, D.D., et al., *Systematic review highlights difficulty interpreting diverse clinical outcomes in abnormal uterine bleeding trials.* J Clin Epidemiol, 2011. **64**(3): p. 293-300.

66. Rodgers, S., et al., *Exploring the outcomes in studies of primary frozen shoulder: is there a need for a core outcome set?* Quality of Life Research, 2014. **23**(9): p. 2495-2504.

67. Rogozinska, E., et al., *Variations in reporting of outcomes in randomized trials on diet and physical activity in pregnancy: A systematic review.* J Obstet Gynaecol Res, 2017. **43**(7): p. 1101-1110.

68. Ronsch, H., et al., *Which outcomes have been measured in hand eczema trials? A systematic review.* Contact Dermatitis, 2019. **80**(4): p. 201-207.

69. Ross, A.R. and N.J. Hall, *Outcome reporting in randomized controlled trials and systematic reviews of gastroschisis treatment: a systematic review.* J Pediatr Surg, 2016. **51**(8): p. 1385-9.

70. Ross, A., et al., *A systematic review of outcomes in postoperative pain studies in paediatric and adolescent patients: towards development of a core outcome set.* Anaesthesia, 2018. **73**(3): p. 375-383.

71. Rubin, T., et al., *Systematic review of outcome measures in pediatric eosinophilic esophagitis treatment trials.* Allergy Asthma Clin Immunol, 2016. **12**(1): p. 45.

72. Sadownik, L.A., P.J. Yong, and K.B. Smith, *Systematic Review of Treatment Outcome Measures for Vulvodynia.* J Low Genit Tract Dis, 2018. **22**(3): p. 251-259.

73. Sautenet, B., et al., *Strong heterogeneity of outcome reporting in systematic reviews.* J Clin Epidemiol, 2016. **75**: p. 93-9.

74. Sautenet, B., et al., *Range and Consistency of Outcomes Reported in Randomized Trials Conducted in Kidney Transplant Recipients: A Systematic Review.* Transplantation, 2018. **102**(12): p. 2065-2071.

75. Sautenet, B., et al., *Scope and Consistency of Outcomes Reported in Randomized Trials Conducted in Adults Receiving Hemodialysis: A Systematic Review.* Am J Kidney Dis, 2018. **72**(1): p. 62-74.

76. Sharif, M.O., et al., *A systematic review of outcome measures used in clinical trials of treatment interventions following traumatic dental injuries.* Dent Traumatol, 2015. **31**(6): p. 422-8.

77. Simpson, R.C., K.S. Thomas, and R. Murphy, *Outcome measures for vulval skin conditions: a systematic review of randomized controlled trials.* Br J Dermatol, 2013. **169**(3): p. 494-501.

78. Sims, M., et al., *Inconsistent selection of outcomes and measurement devices found in shoulder arthroplasty research: An analysis of studies on ClinicalTrials.gov.* PLoS ONE, 2017. **12**(11): p. no pagination.

79. Singendonk, M.M.J., et al., *Variations in Definitions and Outcome Measures in Gastroesophageal Reflux Disease: A Systematic Review.* Pediatrics, 2017. **140**(2).

80. Sinha, I.P., P.R. Williamson, and R.L. Smyth, *Outcomes in clinical trials of inhaled corticosteroids for children with asthma are narrowly focussed on short term disease activity.* PLoS One, 2009. **4**(7): p. e6276.

81. Smail-Faugeron, V., et al., *Development of a core set of outcomes for randomized controlled trials with multiple outcomes--example of pulp treatments of primary teeth for extensive decay in children.* PLoS One, 2013. **8**(1): p. e51908.

82. Smith, P.P., et al., *Outcomes in prevention and management of miscarriage trials: a systematic review.* BJOG, 2019. **126**(2): p. 176-189.

83. Taverny, G., et al., *Outcomes used in randomised controlled trials of nutrition in the critically ill: a systematic review.* Critical Care, 2019. **23**.

84. Tirlapur, S.A., et al., *Variations in the reporting of outcomes used in systematic reviews of treatment effectiveness research in bladder pain syndrome.* Eur J Obstet Gynecol Reprod Biol, 2014. **180**: p. 61-7.

85. Townsend, R., et al., *Variation in outcome reporting in randomized controlled trials of interventions for prevention and treatment of fetal growth restriction.* Ultrasound Obstet Gynecol, 2019. **53**(5): p. 598-608.

86. Tsichlaki, A. and K. O'Brien, *Do orthodontic research outcomes reflect patient values? A systematic review of randomized controlled trials involving children.* American Journal of Orthodontics and Dentofacial Orthopedics, 2014. **146**(3): p. 279-285.

87. Tsichlaki, A., et al., *A scoping review of outcomes related to orthodontic treatment measured in cleft lip and palate.* Orthodontics & Craniofacial Research, 2017. **20**(2): p. 55-64.

88. van Tol, R.R., et al., *Towards a core outcome set for hemorrhoidal disease-a systematic review of outcomes reported in literature.* International Journal of Colorectal Disease, 2018. **33**(7): p. 849-856.

89. Whistance, R.N., et al., *A systematic review of outcome reporting in colorectal cancer surgery.* Colorectal Disease, 2013. **15**(10): p. e548-e560.

90. Whitehead, L., et al., *A systematic review of the outcomes reported in cardiac arrest clinical trials: the need for a core outcome set.* Resuscitation, 2015. **88**: p. 150-7.

91. Wilkinson, J., et al., *No common denominator: a review of outcome measures in IVF RCTs.* Human Reproduction, 2016. **31**(12): p. 2714-2722.

92. Wittkop, L., et al., *Methodological issues in the use of composite endpoints in clinical trials: examples from the HIV field.* Clinical Trials, 2010. **7**(1): p. 19-35.

93. Young, A.E., et al., *Systematic review of clinical outcome reporting in randomised controlled trials of burn care.* Bmj Open, 2019. **9**(2).
