# Supplementary material for: Methods used to assess outcome consistency in clinical studies: A literature-based evaluation
Source: PLoS One. 2020 Jul 8;15(7):e0235485. doi: 10.1371/journal.pone.0235485 (PMC7343158; doi:10.1371/journal.pone.0235485)
Supplement: S4 Table — (DOCX) [file pone.0235485.s006.docx]

**S4 Table** Details of identification of clinical study reports in the evaluation studies of outcome consistency

| **No*** | **Was a language limit applied?** | **Number of search databases** | **Type of covered database(s)?** | **Was a time limit applied?** | **Was application of time limit explained?** | **Use of clinical trials register(s)?** | **Search for Grey literature** | **Other sources?** |
| --- | --- | --- | --- | --- | --- | --- | --- | --- |
| 1 | No | 14 | G&Sp | No | N/A | Yes | Yes | UpToDate.com, Cochrane Methodology Register |
| 2 | No | 7 | G&Sp | Yes | Relevance to research and/or clinical practice | No | No | No |
| 3 | No | 5 | G&Sp | Yes | Not given | NK | NK | Not declared |
| 4 | EN_only | 4 | G&Sp | Yes | Introduction of CONSORT statement | Yes | No | No |
| 5 | No | 2 | G | Yes | relevance to research and/or clinical practice | NK | NK | Not declared |
| 6 | EN_only | 4 | G&Sp | Yes | Introduction of CONSORT statement | NK | NK | Not declared |
| 7 | EN_only | 5 | G | Yes | Introduction of CONSORT statement | NK | NK | Not declared |
| 8 | No | 8 | G | No | N/A | NK | NK | Not declared |
| 9 | No | 1 | Sp | Unclear | N/A | NK | NK | Not declared |
| 10 | No | 4 | G | Yes | Relevance to research and/or clinical practice | Yes | No | No |
| **No*** | **Was a language limit applied?** | **Number of search databases** | **Type of covered database(s)?** | **Was a time limit applied?** | **Was application of time limit explained?** | **Use of clinical trials register(s)?** | **Search for Grey literature** | **Other sources?** |
| 11 | No | n/a | n/a | Yes | Not given | No | No | Top rank journals according to Thomson Reuters 2011 (NEJM, Lancet, J Am Med Ass, Pediatrics, Am J Resp Crit Care Med Crit Care Med, Int Care Med, and Ped Crit Care Med) |
| 12 | EN_only | 2 | G | Yes | Relevance to research and/or clinical practice | NK | NK | Not declared |
| 13 | No | 3 | G | Unclear | N/A | No | No | The references of the included articles |
| 14 | No | 5 | G | Unclear | N/A | No | No | The references of the included articles |
| 15 | EN_only | 2 | G | Yes | Not given | NK | NK | Not declared |
| 16 | EN_only | 2 | G | Yes | Not given | NK | NK | Not declared |
| 17 | EN_only | 1 | G | Yes | Not given | NK | NK | Ten journals in obstetrics and gynaecology from 2008 Institute for Scientific Information rankings |
| 18 | EN_only | 2 | G | Yes | Not given | NK | NK | Not declared |
| 19 | EN_only | 3 | G | No | N/A | NK | NK | Not declared |
| **No*** | **Was a language limit applied?** | **Number of search databases** | **Type of covered database(s)?** | **Was a time limit applied?** | **Was application of time limit explained?** | **Use of clinical trials register(s)?** | **Search for Grey literature** | **Other sources?** |
| 20 | EN_only | 4 | G | No | N/A | NK | NK | Not declared |
| 21 | EN_GER | 3 | G | No | N/A | NK | NK | Not declared |
| 22 | No | n/a | n/a | Yes | Not given | Yes | No | No |
| 23 | No | 1 | G | No | N/A | NK | NK | Not declared |
| 24 | No | 5 | G&Sp | No | N/A | NK | NK | Not declared |
| 25 | No | n/a | n/a | Unclear |  | No | No | Cochrane Reviews |
| 26 | No | 3 | G | No | N/A | NK | NK | Not declared |
| 27 | EN_only | 5 | G&Sp | NR | N/A | Yes | No | No |
| 28 | No | n/a | n/a | No | N/A | No | No | The updated Cochrane syst rev from 2010 |
| 29 | No | 3 | G | Yes | Not given | No | Yes | Google, Google Scholar, Websites of major dental journals, the reference lists of five recently published systematic reviews on the topic peri-implantitis treatment |
| 30 | No | 4 | G&Sp | No | N/A | No | No | The references of the included articles |
| **No*** | **Was a language limit applied?** | **Number of search databases** | **Type of covered database(s)?** | **Was a time limit applied?** | **Was application of time limit explained?** | **Use of clinical trials register(s)?** | **Search for Grey literature** | **Other sources?** |
| 31 | No | 7 | G&Sp | Yes | Not given | No | No | The Patient-reported Health Instruments (PHI) website |
| 32 | EN_only | 8 | G&Sp | Yes | Not given | No | No | A hand search of specialist journals and reference lists of included papers |
| 33 | EN_only | 6 | G&Sp | Yes | Not given | No | No | The search was supplemented by hand searching of abstracts from international meetings in the past 5 years |
| 34 | EN_PT_SP | 6 | G&Sp | Yes | Not given | No | No | The references of the included articles |
| 35 | EN_only | 4 | G&Sp | Yes | Limited resources | Yes | No | The references of the included articles |
| 36 | EN_only | 2 | G | No | N/A | Yes | No | Google Scholar |
| 37 | No | 4 | G&Sp | Yes | Relevance to research and/or clinical practice | NK | NK | Not declared |
| 38 | No | n/a | n/a | Yes | Not given | Yes | No | No |
| 39 | EN_GER_FR_TUR | 1 | G | No | N/A | No | No | The references of the included articles |
| 40 | No | 3 | G | No | N/A | NK | NK | Not declared |
| **No*** | **Was a language limit applied?** | **Number of search databases** | **Type of covered database(s)?** | **Was a time limit applied?** | **Was application of time limit explained?** | **Use of clinical trials register(s)?** | **Search for Grey literature** | **Other sources?** |
| 41 | No | 4 | G | No | N/A | NK | NK | Not declared |
| 42 | EN_only | 3 | G | No | N/A | NK | NK | Not declared |
| 43 | EN_only | 4 | G&Sp | No | N/A | No | No | The references of the included articles |
| 44 | No | n/a | n/a | Yes | Not given | No | No | Journal of Clinical Periodontolog, Journal of Periodontal Research, and Journal of Periodontology |
| 45 | EN_only | 1 | Sp | Yes | Not given | NK | NK | Not declared |
| 46 | EN_only | 1 | G | No |  | No | Yes | Conference abstracts |
| 47 | EN_only | 3 | G&Sp | Yes | Not given | No | No | The reference list of a Cochrane review on relevant topic |
| 48 | EN_only | 3 | G | No |  | NK | NK | Not declared |
| 49 | EN_only | 1 | G | No |  | NK | NK | Not declared |
| 50 | No | 3 | G | No |  | Yes | No | No |
| 51 | EN_only | 3 | G | No |  | No | No | The references of the included articles |
| 52 | EN_only | 4 | G&Sp | Yes | Relevance to research and/or clinical practice | No | No | The references of the included articles |
| **No*** | **Was a language limit applied?** | **Number of search databases** | **Type of covered database(s)?** | **Was a time limit applied?** | **Was application of time limit explained?** | **Use of clinical trials register(s)?** | **Search for Grey literature** | **Other sources?** |
| 53 | EN_GER | 8 | G&Sp | No |  | No | No | The references of the included articles |
| 54 | EN_only | 5 | G&Sp | No |  | No | Yes | The references of the included articles |
| 55 | No | 2 | G | Yes | Introduction of CONSORT statement | NK | NK | Not declared |
| 56 | No | 3 | G | Yes | Introduction of CONSORT statement | NK | NK | Not declared |
| 57 | EN_only | 3 | G | No |  | No | No | Backward & forward citation tracking of relevant articles; hand searching of all titles and relevant abstracts of studies published in the “British Journal of Surgery” and “Annals of Surgery” (20yrs period) |
| 58 | EN_only | 3 | G&Sp | Yes | Relevance to research and/or clinical practice | Yes | No | No |
| 59 | EN_only | 1 | G | Yes | Not given | NK | NK | Not declared |
| 60 | No | 4 | G&Sp | No |  | Yes | No | The references of the included articles |
| 61 | EN_only | 2 | G | Yes | Not given | NK | NK | Not declared |
| 62 | EN_only | 6 | G&Sp | No |  | No | No | The references of the included articles |
| **No*** | **Was a language limit applied?** | **Number of search databases** | **Type of covered database(s)?** | **Was a time limit applied?** | **Was application of time limit explained?** | **Use of clinical trials register(s)?** | **Search for Grey literature** | **Other sources?** |
| 63 | No | 3 | G | No |  | NK | NK | Not declared |
| 64 | EN_only | 4 | G&Sp | Yes | Relevance to research and/or clinical practice | No | No | The references of the included articles |
| 65 | EN_only | 1 | G | No |  | No | No | The references of the included articles |
| 66 | No | 2 | G | Yes | Update of prior syst rev | No | No | Prior syst rev |
| 67 | No | 4 | G&Sp | Yes | Not given | NK | NK | Not declared |
| 68 | No | 2 | G | Yes | Not given | Yes | No | The references of the included articles |
| 69 | No | 3 | G | No |  | NK | NK | Not declared |
| 70 | No | n/a | n/a | Yes | Not given | Yes | No | No |
| 71 | EN_only | 4 | G&Sp | Yes | Not given | Yes | No | No |
| 72 | EN_only | 5 | G&Sp | No |  | NK | NK | Not declared |
| 73 | EN_only | 2 | G | NK |  | NK | NK | Not declared |
| 74 | EN_only | 1 | G | No |  | Yes | No | No |
| 75 | EN_only | 1 | G | No |  | Yes | No | No |
| 76 | No | 4 | G | No |  | NK | NK | Not declared |
| 77 | EN_only | 3 | G | No |  | NK | NK | Not declared |
| 78 | No | n/a | n/a | Yes | Not given | Yes | No | No |
| **No*** | **Was a language limit applied?** | **Number of search databases** | **Type of covered database(s)?** | **Was a time limit applied?** | **Was application of time limit explained?** | **Use of clinical trials register(s)?** | **Search for Grey literature** | **Other sources?** |
| 79 | EN_only | 4 | G | No |  | No | No | The references of the included articles |
| 80 | No | 1 | G | Yes | Not given | No | No | Journals and conference proceedings |
| 81 | No | 6 | G&Sp | No |  | Yes | Yes | The references of the included articles |
| 82 | No | 4 | G&Sp | Unclear |  | No | Yes | The references of the included articles |
| 83 | EN_FR | 2 | G | Yes | Not given | Yes | No | The references of the included articles |
| 84 | No | 6 | G&Sp | No |  | No | Yes | The references of included studies, and conference proceedings of the International Continence Society |
| 85 | EN_only | 3 | G | No |  | No | No | The references of the included articles |
| 86 | No | 5 | G&Sp | Yes | Not given | No | No | The references of included studies and trials identified in recently published Cochrane syst rev |
| 87 | No | 7 | G&Sp | Yes | Not given | Yes | No | The references of the included articles |
| **No*** | **Was a language limit applied?** | **Number of search databases** | **Type of covered database(s)?** | **Was a time limit applied?** | **Was application of time limit explained?** | **Use of clinical trials register(s)?** | **Search for Grey literature** | **Other sources?** |
| 88 | No | 3 | G | Yes | Having a representative sample | No | No | The references of the included articles |
| 89 | EN_only | 3 | G | Yes | Relevance to research and/or clinical practice | No | No | The websites of relevant medical journals, reference lists of included studies |
| 90 | EN_only | 4 | G | Yes | Not given | NK | NK | Not declared |
| 91 | No | 1 | G&Sp | Yes | Limited resources | No | No | E-mail alerts from various journals and other databases; conference abstracts and journals |
| 92 | No | 1 | G | Yes | Not given | No | No | Conference on Retroviruses and Opportunistic Infections, Interscience Conference on Antimicrobial Agents and Chemotherapy, the International AIDS Conference and the European AIDS Conference during the same time period |
| 93 | EN_only | 4 | G | Yes | Relevance to research and/or clinical practice | NK | NK | Not declared |

**Numbers correspond with the studies listed in Appendix 5*

*N/A, not applicable; Syst rev, systematic review; NK, not known*

*Abbreviations in second column: EN_only, publication only in English language; GER, German language; FR, French; TUR, Turkish; PT, Portuguese; SP, Spanish;*

*Abbreviations in fourth column: G, general (e.g. Medline, Embase, Web of Science); Sp, specialist (e.g. Latin American and Caribbean Literature on Health Sciences, The Cumulative Index to Nursing and Allied Health Literature)*
