# Supplementary material for: Methods used to assess outcome consistency in clinical studies: A literature-based evaluation
Source: PLoS One. 2020 Jul 8;15(7):e0235485. doi: 10.1371/journal.pone.0235485 (PMC7343158; doi:10.1371/journal.pone.0235485)
Supplement: Attachment — Submitted filename: 20Jun01 Response.docx [file pone.0235485.s007.docx]

| **No.** | **Comment** | **Response** |
| --- | --- | --- |
| **Reviewer #1:** | | |
| 1 | The authors propose to evaluate the quality of the methods used in studies on outcomes consistencies. The topic is important and has not been investigated. However a few point could be improved | We thank the reviewer for the recognition of the importance of this topic. |
|  | **Major comments** |  |
| 2 | 1) The author declare that their study was not a systematic review and reported having searched in the “COMET Database”. It is not clear what this COMET database is and the reference 8 does not mention it clearly either (the weblink of reference 8 is broken: <http://www.comet-initiative.org/studies/search>) It is time consuming for the reader to find out how this COMET database is provided (medline and scopus mainly?).  In the method section, the authors should explain with a few sentences how the COMET database is fuelled. | We agree with the reviewer that we had not adequately described the COMET database and have now addressed this shortcoming by adding the following description in the methods:  “We searched the COMET database from its inception to June 2018; the search was updated in May 2019. (8) The COMET database is an annually updated repository of the international COS literature based on the systematic searches run in MEDLINE, SCOPUS, and Cochrane Methodology Register. (9) |
| 3 | 2) The title of the article does not give any idea about the method they used. We propose to add more precision about the design of their study as a scoping review see the reference of the Joanna Briggs Institute (<https://wiki.joannabriggs.org/display/MANUAL/Chapter+11%3A+Scoping+reviews>) or of the PRISMA group (<http://www.prisma-statement.org/Extensions/ScopingReviews>)  “Evaluation of methods in studies of outcomes consistency in clinical studies: a scoping review” | We thank the reviewer for the suggestion. However, our work was not set out to be a scoping review; thus we feel that labelling its design this way would be inappropriate. According to our protocol (see the PROSPERO registration) the design was described as “exploratory study” and was developed in the spirit of meta-research; discipline aiming to evaluate and improve research practices (Ioannidis et al. 2015 PLoS Biol. 13(10): e1002264). We have now added the information about the study design in the title as follows: “Methods to assess outcome consistency in clinical research: literature-baseded evaluation” |
| 4 | 3) At the end of the discussion, the author recommend not to use the term “systematic review” when studies are not: “However, we discourage labelling for this type of evaluation, as “systematic review” instead, proposing alternative terms such as an “evaluation of outcomes” or “outcome mapping”.” We also suggest to use the term of “scoping review” in order to standardized the terminology according to PRISMA and the Joanna Briggs institute. | We agree that a scoping review may be another alternative and have amended the last sentence in our final paragraph as follows:  “However, we discourage labelling for this type of evaluation, as “systematic review.” Instead, we propose terms such as an “evaluation of outcomes”, “outcome mapping.” Alternatively, authors ma choose to use an established approach, such as “scoping review”, and adopt the appropriate methodological and reporting standards (34, 35).” |
|  | **Minor comments** |  |
| 5 | Table 1: we do not understand what “total number of participants per review” does mean. Who are these participants? Is it the number of reviewers involved in each study or the number of patients included in the studies evaluated by the review? In case of the later, how is it relevant for the current study? | On reflection we agree with the reviewer and have now removed this information from Table 1. |
| **Reviewer #2** | | |
| 6 | The abstract is not fully understandable without reading the main text. You should state concisely which type of studies are included in your review (e.g. synthesis studies evaluating outcome consistency in clinical studies).  Please do not use undefined abbreviations (AMSTAR). | We have now amended the abstract and checked the manuscript for consistency in the used terminology referring to included studies as “evaluations of outcome consistency in clinical studies”  AMSTAR abbreviation has been explained in the abstract as follows: “The methods were assessed using a subset of A MeaSurement Tool to Assess systematic Reviews (AMSTAR) 2 items (1-9) and additional outcome-specific questions.” |
| 7 | Please provide a brief description of AMSTAR in the main text. | We have now added a following description in the methods section: “In order to examine adoption of systematic review methodology, all studies (regardless of declared study design) were assessed against a tailored subset of A MeaSurement Tool to Assess systematic Reviews (AMSTAR) 2 items (23) we felt were relevant to evaluated studies (S1 Table). AMSTAR was designed as a practical critical appraisal instrument enabling a rapid and reproducible quality assessments of systematic reviews.” |
| 8 | Table 1: please move the notes into the table since they include relevant information | The notes have been now incorporated into the main body of Table 1. |
| 9 | Table 3: it is not understandable why denominators differ across the rows (90 or 93). | The explanation of the difference is provided at the bottom of the table and has been rewritten for clarity: *“*Three reports (3/93) of full core outcome sets were excluded from some assessments due to none applicability*  *(for details see S3 Table)”.* We also moved asterisks to the column heading (n/N*). |
| 10 | Tables (in general): when you use the word "studies", please verify whether it is easy to distinguish between studies included in your review and clinical studies included in the synthesis studies you examined | We have now amended all tables to improve their clarity according to the reviewer’s suggestion. |
| 11 | Please check all tables and figure captions and see if they can be more informative of the underlying content. | We have now checked all tables and figures to improve their clarity according to this suggestion. |
| 12 | Please discuss any practical implications of your work (such as improvements in clinical research and practice). | The practical implications of our work are discussed in the final paragraph of our discussion, notably that authors of these studies need to be transparent in reporting their methods and that they should not label such evaluations as systematic reviews if they do not fully adhere to stringent systematic review methodologies.  Although we do not feel that our work has any direct impact on clinical practice; we hope that it will improve research practice relating to core outcome set development and reporting of such research. |
| 13 | Please identify any health area that requires more attention in future research. | As our work focuses on the methods rather than a specific clinical issue and is applicable across all health areas. We have recorded and reported the health areas in which evaluations of outcome consistency were carried out, however, we have not compared methodological practices between different health areas. |
