# Supplementary material for: Methods used to assess outcome consistency in clinical studies: A literature-based evaluation
Source: PLoS One. 2020 Jul 8;15(7):e0235485. doi: 10.1371/journal.pone.0235485 (PMC7343158; doi:10.1371/journal.pone.0235485)
Supplement: Attachment — Submitted filename: 20Jun14 Response to reviewers.docx [file pone.0235485.s008.docx]

**Response to reviewers**

| **No.** | **Comment** | **Response** |
| --- | --- | --- |
| **Reviewer #2** | | |
| 1 | Thanks for addressing my previous comments. I have few additional minor suggestions: | We thank the reviewer for his further suggestions. We have reviewed all the points and amended the manuscript accordingly. |
| 2 | 1) replace "evaluations of outcome consistency" with "evaluation studies of outcome consistency"  2) if possibile, include such denomination among the keywords | The term "evaluations of outcome consistency" has been changed to "evaluation studies of outcome consistency" and listed the term among the keywords. |
| 3 | 3) check Table 1 caption (it should be outcome "consistency") | The caption of Table 1 has been corrected to: “Characteristics of included evaluations of outcome consistency” |
| 4 | 4) Table 3 caption: also in this case I would specify that methods are those of the included studies | The caption of Table 3 has been changed to: “Methods used to identify and assess outcomes in evaluation studies of outcome consistency” |
| 5 | 5) Table 3: the third category ("when the distinction was made..") is a sub-category of the second one, and the way of reporting should reflect this | The category and subcategories have been amended as follows:  When the distinction between the type of outcome(s) was made:   - The primary outcome(s) was clearly specified as a primary - The primary outcome(s) was clearly specified as a primary or used in the power calculation - The primary outcome(s) was identified using other measures (e.g. outcome mentioned in the trial title, first reported) - There were no details of how the primary outcome(s) was identified |
| 6 | 6) Table 3: the note is still unclear (you should fully understand the table without looking at supplementary files) | The note has been amended as follows:  “Three reports (3/93) of full core outcome sets were excluded from some assessments (N = 90) as they focused only on providing a list of identified outcomes without reporting more details (see S3 Table)” |
| 7 | 7) S3 Table caption: it should be "detailed characteristics" instead of "detail characteristics" | The caption of S3 Table has been changed to: “Detailed characteristics of included evaluation studies of outcome consistency” |
| 8 | 8) S4 Table caption is unclear: do you mean the clinical studies, or the evaluation studies that you assessed? | The caption of S4 Table has been changed to: “Details of identification of clinical study reports in the evaluation studies of outcome consistency” |
